# The genetic architecture of resistance to virus infection in *Drosophila*

**DOI:** 10.1111/mec.13769

**Published:** 2016-08-26

**Authors:** Rodrigo Cogni, Chuan Cao, Jonathan P. Day, Calum Bridson, Francis M. Jiggins

**Affiliations:** ^1^ Department of GeneticsUniversity of CambridgeCambridgeCB2 3EHUK; ^2^ Department of EcologyUniversity of São PauloSão Paulo05508‐900Brazil

**Keywords:** genomics/proteomics, insects, parasitology, quantitative genetics, species interactions

## Abstract

Variation in susceptibility to infection has a substantial genetic component in natural populations, and it has been argued that selection by pathogens may result in it having a simpler genetic architecture than many other quantitative traits. This is important as models of host–pathogen co‐evolution typically assume resistance is controlled by a small number of genes. Using the *Drosophila melanogaster* multiparent advanced intercross, we investigated the genetic architecture of resistance to two naturally occurring viruses, the sigma virus and DCV (Drosophila C virus). We found extensive genetic variation in resistance to both viruses. For DCV resistance, this variation is largely caused by two major‐effect loci. Sigma virus resistance involves more genes – we mapped five loci, and together these explained less than half the genetic variance. Nonetheless, several of these had a large effect on resistance. Models of co‐evolution typically assume strong epistatic interactions between polymorphisms controlling resistance, but we were only able to detect one locus that altered the effect of the main effect loci we had mapped. Most of the loci we mapped were probably at an intermediate frequency in natural populations. Overall, our results are consistent with major‐effect genes commonly affecting susceptibility to infectious diseases, with DCV resistance being a near‐Mendelian trait.

## Abstract 2

see also the Perspective by Magalhães and Sucena

## Introduction

Variation in susceptibility to infectious disease often has a substantial genetic component in natural populations, including plants (Thompson & Burdon 1992), invertebrates (Lazzaro *et al*. 2004; Bennett *et al*. 2005) and humans (Cooke & Hill 2001). This variation is of great importance in allowing the selective breeding of disease‐resistant forms in agriculture and in understanding the incidence of infection within populations. Additionally, studying the causes of variation in susceptibility to infectious diseases provides insights into co‐evolution and the evolution of resistance to pathogens (Sorci *et al*. 1997).

The processes that maintain genetic variation in susceptibility in populations are still a matter for debate. Because pathogens are an important selective force in the wild, there is probably to be strong natural selection on this variation in populations. This can be positive selection that drives resistance alleles through fixation (Woolhouse *et al*. 2002; Bangham *et al*. 2007; Magwire *et al*. 2011). In this scenario, variation may result from the continual input of new resistance alleles into populations by mutation, and because the direction of selection continually changes as new pathogens appear (Woolhouse *et al*. 2005), existing pathogens evolve to escape host defences (Woolhouse *et al*. 2002), or environmental conditions change. However, most theoretical attention has been paid to models in which co‐evolution between hosts and pathogens results in negative frequency‐dependent selection that can maintain both resistant and susceptible alleles of a gene in populations (Clark 1976; Stahl *et al*. 1999; Woolhouse *et al*. 2002). This process is of particular interest as it may favour the evolution of sexual reproduction and recombination (Jaenike 1978). These models make strong assumptions about the genetic architecture of resistance – typically that a small number of major‐effect loci control host resistance and that there are strong epistatic interactions between loci (Tellier & Brown 2007). Understanding the maintenance of genetic variation in susceptibility therefore requires an understanding of the genetic architecture of resistance – the number of genes involved, their effect sizes and their interactions.

Quantitative traits typically have a complex genetic basis, and in most cases, we have a poor understanding of the genome positions, phenotypic effects and population frequencies of the underlying genetic variants contributing to phenotypic variation (Zuk *et al*. 2012). In human association studies, a combination of larger sample sizes and the use of resequencing rather than genetic markers means that this is beginning to change, but there is still a substantial discrepancy between the heritability estimates of a trait and the amount of heritable variation accounted for by all variants identified (Manolio *et al*. 2009). Possible explanations of this ‘missing heritability’ include widespread allelic heterogeneity (multiple independent effects segregating at each causative locus) (Thornton *et al*. 2013; King *et al*. 2014), widespread epistasis (Huang *et al*. 2012; Zuk *et al*. 2012; Mackay 2014), many very small effect loci (Yang *et al*. 2010; Rockman 2012) and large numbers of rare alleles of large effect (Bansal *et al*. 2010; Thornton *et al*. 2013).

Susceptibility to disease is a complex trait whose genetic architecture has been extensively investigated over the last decade by genomewide association studies (GWAS) (Visscher *et al*. 2012). The majority of these focused on noncommunicable diseases in humans, and here, the polymorphisms identified usually have small effects and can explain only a small fraction of heritability (Pritchard 2001). A possible reason for this is that the variation results from new mutations that increase susceptibility, and therefore, moderate‐ or large‐effect alleles will be either removed from the population or kept at a low frequency by purifying selection (Pritchard 2001). However, the genetic architecture of susceptibility to infectious diseases may be different (Hill 2012; Magwire *et al*. 2012). Major‐effect polymorphisms that decrease susceptibility to infection have been identified in many organisms by both GWAS and classical QTL and linkage mapping (Bangham *et al*. 2007, 2008; Wilfert & Schmid‐Hempel 2008; Magwire *et al*. 2011, 2012; Hill 2012; Cao *et al*. 2016). The ever‐changing selection pressures exerted by pathogens may drive new major‐effect resistance alleles up in frequency by positive selection, while negatively frequency‐dependent selection may maintain existing variation (Stahl *et al*. 1999; Magwire *et al*. 2011). This suggests that natural selection may increase the frequency of major‐effect alleles in populations, causing the genetic architecture of susceptibility to infectious diseases to be simpler than is the case for noncommunicable diseases (Hill 2012; Magwire *et al*. 2012).

*Drosophila melanogaster* is an excellent model to study the genetic architecture of susceptibility to pathogens. Unlike in humans, studies can take advantage of controlled and highly replicated experimental infections on genetically identical flies. Natural populations of *D. melanogaster* are infected by a variety of viruses, including DCV (Drosophila C virus) and the sigma virus. DCV is a single‐stranded positive‐sense RNA virus in the family Dicistroviridae (Christian 1987; Arnold *et al*. 2013). The sigma virus is a single‐stranded negative‐sense RNA virus in the rhabdovirus family that is a specialist on *D. melanogaster* (Brun & Plus 1980; Longdon *et al*. 2012). While DCV is transmitted horizontally, the sigma virus is only transmitted vertically from parent to offspring (Brun & Plus 1980; Christian 1987). DCV is a very virulent virus, with infection causing a depression of the metabolic rate followed by death (Arnold *et al*. 2013). By contrast, the sigma virus does not kill flies, but it is thought to cause a approximately 20% reduction in their fitness (Yampolsky *et al*. 1999; Longdon *et al*. 2012; Wilfert & Jiggins 2013).

We have previously used whole‐genome association studies to investigate genetic variation in susceptibility to DCV and the sigma virus. For the sigma virus, we identified two major‐effect polymorphisms in the genes *CHKov1* and *ref(2)P* associated with resistance, and these together explain 37% of the genetic variance in the population (Contamine *et al*. 1989; Magwire *et al*. 2011; #37, Magwire *et al*. 2012; #36). For DCV, we identified a single major‐effect gene called *Pastrel* that explains 47% of the genetic variance in resistance (Magwire *et al*. 2012). Although these association studies were very successful in explaining a large proportion of genetic variation compared to most studies on the genetic basis of complex traits, there is still a large proportion of genetic variation not explained, and the causes of this missing heritability are unknown.

To address this problem, we used the *Drosophila melanogaster* multiparent advanced intercross, known as the DSPR (http://FlyRILs.org) (King *et al*. 2012; Long *et al*. 2014). To detect rare or small effect variants (Manolio *et al*. 2009), multiparent advanced intercross mapping panels have been proposed as a simpler and less expensive approach than the recently popular studies using very large‐scale exome resequencing (Do *et al*. 2012; Mirabello *et al*. 2014). These panels have been developed for mouse (Churchill *et al*. 2004), *Arabidopsis* (Kover *et al*. 2009; Huang *et al*. 2012; #26), maize (Buckler *et al*. 2009) and *Drosophila* (King *et al*. 2012). They are formed by crossing several inbred founder lines for multiple generations to create a population whose genomes are fine‐scale mosaics of the original founder lines’ genomes. The DSPR was created by mixing two groups of eight inbred founder lines in two populations and allowing them to interbreed for 50 generations. Flies from these populations were then inbred to create over 1700 recombinant inbred lines (RILs) (King *et al*. 2012). Complete genome sequence data for the founder lines are available. A high density of molecular markers is scored in each RIL, allowing each position in the genome to be probabilistically assigned to one of the founder lines. Compared to classical quantitative trait locus (QTL) mapping, these resources provide a much higher resolution of QTL positions and, by being founded by multiple genotypes, allow estimates of the frequency of alleles at QTL. The high resolution of the QTL is important, as otherwise what appears to be a single major‐effect QTL often proves to be multiple linked loci (Mackay *et al*. 2009).

Using a multiparent advanced intercross has several advantages compared to our published work on virus resistance that used whole‐genome association studies (Magwire *et al*. 2012). This previous work used a panel of fly lines (the DGRP lines) from a population in North America that had been inbred and had their genomes resequenced (Magwire *et al*. 2012). We were limited to *c*. 150 lines, and after corrections for multiple testing, we could only had the statistical power to identify common major‐effect variants. The first advantage of the DSPR is that we have the statistical power to detect new variants with smaller effect sizes. In our previous work, we tested associations between *c*. 2.5 million SNPs and the phenotype, needing severe correction for multiple testing. With the DSPR, we can test the effect of local haplotypes of a few cM in size on the phenotype, greatly reducing the number of tests. In addition, in this study we used more than 800 lines, giving many more independent observations for each site in the genome. Second, this increase in statistical power gives us greater ability to detect additional loci that epistatically modify the effects of the QTL we identify. Third, in the DSPR it is possible to identify variants that are rare in natural populations, as rare alleles present in the eight founders will be pushed to intermediate frequencies (on average 12.5%). Because the panel is founded by eight parents, most of the rare variants segregating in nature will not be included meaning that some important natural polymorphisms may be missing from the lines. However, as we find that the DSPR panel has a similar level of genetic variation as natural populations, if this variation is caused by rare alleles of large effect then some of these alleles have been captured in the genomes of the eight founders. This is to be expected, as if rare variants contribute substantially to genetic variation in natural populations, there are probably to be many of them. Finally, another difference of the DSPR from our previous work is that the parental lines are sampled from around the world. This will allow the identification of new variants that are not found in the North Carolina population we studied before, although we would caution that coadapted gene complexes may have been broken up in this process. This is important, as the prevalence and genotype of pathogens commonly vary geographically, which may alter patterns of genetic variation.

In this study, we found extensive genetic variation among the DSPR lines in resistance to the sigma virus and DCV. For each virus, we first identified a single major‐effect locus that was previously known to be associated with resistance. After controlling for these loci, we were able to identify additional QTL, several of which had substantial effects on resistance. Furthermore, we found little evidence of epistasis, detecting only a single locus that modified the effects of the QTL. These new QTL provide a list of new candidate genes affecting virus resistance.

## Materials and methods

### Virus production

The Hap23 strain of the sigma virus (Coulon & Contamine 1982) was extracted from an infected line of *D. melanogaster* (EX320). One hundred 15‐day old flies were frozen at −80 °C, homogenized in 1 mL of Ringer's solution and centrifuged twice at 13 000 ***g*** for 30 s at 4 °C. The supernatants from replicated tubes were mixed together, and the extract was then separated in small aliquots and stored at −80 °C. DCV‐C (Jousset *et al*. 1972) was kindly provided by Luis Teixeira (Teixeira *et al*. 2008). It was cultured in *Drosophila melanogaster* DL2 cell culture, and the Tissue Culture Infective Dose 50 (TCID_50_) was calculated by standard protocol (Johnson & Christian 1999; Martinez *et al*. 2014).

### Fly lines

We only used panel B of the DSPR. Recombinant inbred lines were obtained from S. J. Macdonald (King *et al*. 2012) and kept at 25 °C. The original founder lines had been cleaned for *Wolbachia* infection. We tested whether the lines were previously infected with sigma or DCV. For the sigma virus, *c*. 15% of the lines were tested for symptom of sensitivity to CO_2_ as described below; none of flies tested were dead or paralysed after CO_2_ exposure. For DCV, *c*. 10% of the lines were tested by standard qPCR (Martinez *et al*. 2014), and none were infected. We used PCR to genotype the founder lines and selected RILs for polymorphisms in the genes *ref(2)p* and *CHKov1* that have been previously associated with virus resistance. Two flanking universal primers (ref2p‐P1‐F 5′‐CTCACCCAGCTGCACTTGTA‐3′, ref2p‐PS1‐R 5′‐TGTTGCAATCTTTGCGACTC‐3′) and a specific primer for each allele (susceptible allele: ref‐a1‐Forward 5′‐GGATGCCCTCCCAGAATTA‐3′; recessive allele: ref‐a1‐Reverse 5′‐ CGACGCAATRYGGTGTATCC‐3′) were used to genotype *ref(2)p* (Wilfert & Schmid‐Hempel 2008). A forward primer CHK_F (59 CTCTTGGCTCCAAACGTGAC 39) and reverse primer CHK_R (59 AAGGCAAACGACGCTCTT 39) were used to detect the absence of the *Doc1420* element in *CHKov1*. The forward primer Doc1420_F (59 CTTGTTCACATTGTCGCTGAG 39) was used with the reverse primer CHK_R to detect the presence of the *Doc1420* element in *CHKov1* (Magwire *et al*. 2011).

### Resistance assays

The generation prior to virus infection was set up with three males and three females that were allowed to lay eggs for 48 h in a vial with standard cornmeal–agar food. For each line, we injected 20 mated females that were 3–6 days old. For most of the lines, a single vial was used per RIL, and for *c*. of 15% of the lines, a second biological replicate (another vial) was performed. For the sigma virus, a total of 635 lines were used and 94 were replicated. For DCV, a total of 619 lines were used and 107 were replicated. *c*. of 50 vials were infected per day, and for replicated lines, each vial was infected in a different day. For the sigma virus, 69 nL of the virus extract was injected in the abdomen as in Longdon *et al*. (Longdon *et al*. 2011). Injected flies were kept on cornmeal–agar food and assayed for infection 13 days postinjection. Flies were exposed to pure CO_2_ for 15 min at 12 °C and 30 min postexposure flies that were awakened were classified as uninfected and flies that were dead or paralysed were classified as infected. For DCV, females were pricked with DCV suspension as in Longdon *et al*. (Longdon *et al*. 2015) and kept on cornmeal–agar food. Mortality of flies was recorded for 15 days. Flies that died within 24 h were excluded from the analysis as it was assumed that they died from the pricking process.

### QTL mapping

First, we evaluated the repeatability of our resistance assay and estimated the amount of genetic variation in resistance to each virus. For DCV, we fitted in a linear mixed‐effect model. Let *y*_*i,j,k*_ be the mean survival time in days of flies in vial *k* from RIL *j* on injection date *i*:

(eqn1) $$y_{i,j,k}=\beta +{\mathrm{date}}_{i}+{\mathrm{RIL}}_{j}+\varepsilon_{i,j,k}$$

where β is the overall mean survival time, date_*i*_ is a random variable representing the deviation from the overall mean of vials injected on the date *i,* and RIL_*j*_ is a random variable representing the deviation of RIL *j*. ε_*i,j,k*_ is the residual error. For the sigma virus, we fitted a similar model to (1) except the response variable was the proportion of infected flies in a vial. Using the parameters estimated in this model, we calculated the repeatability, *R,* of our assay:

(eqn2) $$R=\frac{\sigma_{\mathrm{RIL}}^{2}}{\sigma_{\mathrm{RIL}}^{2}+\sigma_{\varepsilon }^{2}}$$

where $\sigma_{\mathrm{RIL}}^{2}$ is the between‐RIL variance and $\sigma_{\varepsilon }^{2}$ is the residual variance. Note this does not include $\sigma_{\mathrm{date}}^{2}$ (the between injection‐date variance), so it is repeatability on a single day. *R* and its 95% confidence intervals were estimated using the r package Heritability. For use in QTL analyses below, we estimated the best linear unbiased predictor (BLUP) for the phenotype of each RIL (i.e. a phenotype corrected for the effect of injection date).

The QTL analyses were performed using the r package dsprqtl (http://FlyRILs.org/Tools/Tutorial) (King *et al*. 2012). Following King *et al*. (2012), we regressed our resistance phenotype on the eight founder genotype probabilities at evenly spaced 10‐kb positions across the genome, converting the resulting *F*‐statistic to a LOD score (Broman & Sen 2009). We determined the genomewide significance by permuting the phenotypic data across the lines, repeating the QTL analysis and recording the highest LOD score across the entire genome. We repeated this 2000 times to give a null distribution of the maximum LOD score (Churchill & Doerge 1994). To localize peaks more precisely, we performed interval mapping locally around the main mapped QTL (Lander & Botstein 1989) (Broman & Sen 2009). Using these results, we estimated intervals on the locations of the QTL using both 95% Bayesian credible intervals and a LOD drop of 2.

To identify additional QTL influencing virus resistance, we performed a second analysis that statistically controls for the effects of the main QTL found in the first analysis. To do this, we performed a genome QTL scan where the main QTL from the first analysis was a covariate. For the sigma virus, the first QTL we identified is caused by the gene *ref(2)p*, and here, we know the genetic change that causes resistance (see Results for details). Therefore, we could assign each RIL to either being *ref(2)P* resistant or susceptible (where this was ambiguous from the genotype probabilities, we genotyped the lines by PCR). The first QTL that we identified in the DCV experiment was caused by the gene *pastrel* (*pst;* see Results for details). As the variant in *pst* that causes resistance is unknown, we accounted for the effects of this gene by including the eight *pst* founder genotype probabilities as a covariate. This led to the identification of several additional QTL. To simplify the local interval mapping and estimation of confidence intervals for these additional QTL, we used BLUPs for each RIL accounting for the effects of *ref(2)P* and *pst* rather than including these genes as covariates as was the case in other analyses. To do this, we used the GLM:

(eqn3) $$y_{h,i,j,k}=\beta +{\mathrm{QTL}}_{h}+{\mathrm{date}}_{i}+{\mathrm{RIL}}_{j}+\varepsilon_{i,j,k}$$

where the parameters are the same as model 1 except QTL_*h*_ which is a fixed effect of allele *h* of the *ref(2)P* or *pst* QTL. The model parameters were estimated by REML using the lme function in r.

At each QTL, we assigned the founder alleles to the two most likely allelic classes (‘resistant’ and ‘susceptible’). Following King *et al*. 2012; we first ranked the founder genotype at each QTL according to their mean phenotype. We then split this ranked list into all possible classes (‘resistant’ and ‘susceptible’). We performed anovas for all these different groups and choose the grouping with the highest *F*‐value as the best two‐class partition (King *et al*. 2012). For each RIL, we then calculated the probability that it carried the resistant allele by summing the genotype probabilities of the resistant and susceptible founders.

### Effect sizes of the QTL and analyses of genetic variation

To estimate the proportion of the genetic variance in the mapping population that was explained by each QTL, we compared the between‐RIL variance estimated using mixed models that either included the QTL as a fixed effect (model 3 above, using the eight genotype probabilities as the fixed‐effect QTL_*h*_) or that did not (model 1 above). We compared the change in the between‐RIL variance between the two models to calculate the proportion of genetic variance explained by the QTL. To allow direct comparison of the RIL variances from the two models, we fitted these models using a Bayesian approach with mcmcglmm r package (Hadfield 2010).

To estimate the effect size of each QTL, we again modified model 3. Here, we treated fixed‐effect QTL_*h*_ as the probability of each RIL carrying the resistant allele of the QTL (see above for how this was calculated). We also included all the different QTL that we identified as fixed effects in the same model. Again, the model parameters were estimated using MCMCglmm.

### Epistasis

The first approach we took to detect epistasis was to test for pairwise epistasis between the QTL detected above on the basis of their main effects. Let *y*_*g,h,i,j,k*_ be the phenotype of a vial of flies (mean survival time of DCV‐infected flies or proportion of sigma virus‐infected flies) with allele *g* of QTL1 and allele *h* of QTL2, from vial *k* and RIL *j*, injected on date *i*:

(eqn4) $$\begin{array}{cc} Y_{g,h,i,j,k}=\; & \beta +{\mathrm{QTL}}_{g}+{\mathrm{QTL}}_{h}+{\mathrm{QTL}}_{g}:{\mathrm{QTL}}_{h}\\ & +{\mathrm{date}}_{i}+{\mathrm{RIL}}_{j}+\varepsilon_{g,h,i,j,k} \end{array}.$$

The model parameters are the same as for model 1, except QTL_*g*_ and QTL_*h*_ which are the fixed effects of the two QTL, and QTL_*g*_:QTL_*h*_ which is the epistatic interaction between the QTL (QTL was a categorical variable with two levels: resistant or susceptible). We fitted models by maximum likelihood using the lme function in r.

Loci that epistatically modify the effects of other QTL might not be detectable from their main effects. To identify such QTL that interact with the identified QTL we ran genome scans looking for significant interaction terms at 10 kB intervals across the genome:

(eqn5) $$y_{g,h,k}=\beta +{\mathrm{QTL}}_{g}+{\mathrm{Locus}}_{h}+{\mathrm{QTL}}_{g}:{\mathrm{Locus}}_{h}+\varepsilon_{g,h,k}$$

where *y*_*g,h,k,*_ is the BLUP of the mean phenotype of RIL *k* corrected for the effects of injection date and *ref(2)P/pst* (see above for details; *ref(2)P/pst* not corrected for when these genes were being investigated). β is the overall mean. QTL_*g*_ is a fixed effect of allele *g* of a QTL identified previously (expressed as a probability of being resistant or susceptible). Locus_*h*_ is a fixed effect of the 10‐kB position being tested, expressed as the eight possible genotype probabilities. QTL_*g*_:Locus_*h*_ is the interaction between these terms. ε_*g,h,k*_ is the residual. The model parameters were estimated using the lm function in r. The LOD score for the interaction term was calculated by comparing the likelihood of model 5 to an equivalent model that lacked the interaction term. Then, we used permutations to determine the genomewide significance threshold following the procedure described above. We were not able to do this analysis for the X13 QTL because we only have 18 resistant RIL (BB5 parent) and, therefore, many genotype combinations were rare or missing.

### Data availability

The raw data and scripts used in this study are available at University of Cambridge data repository (https://www.repository.cam.ac.uk/handle/1810/255877). All analyses were carried out in r (3.1.1). The package versions were dsprqtl (2.0‐4), lme4 (1.1‐70), car (2.0‐20), mcmcglmm (2.21), ggplot (2.14.1) and ggplot2 (1.0.1).

## Results

### Extensive genetic variation in resistance to viruses

We inoculated more than 26000 flies with virus in the laboratory. For DCV, we inoculated 14091 flies from 619 RIL, with 107 of these RIL having independent biological replicates (flies from independent vials). As DCV is a highly virulent virus, we measured resistance by recording mortality. For the sigma virus, we injected 12195 flies from 635 RIL, with 94 of these lines having independent biological replicates. As the sigma virus does not kill flies, we recorded the proportion of individuals that became paralysed after exposure to CO_2_, which is a characteristic symptom of infection.

**Figure 1:**
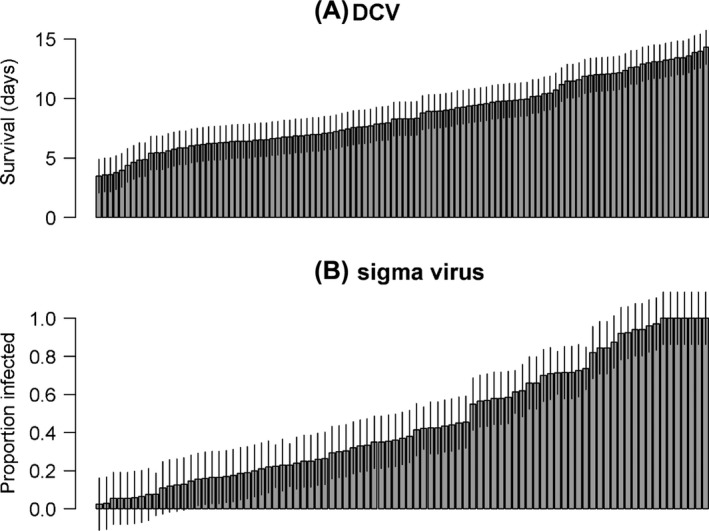
Variation in resistance to DCV (A) and sigma virus (B). Each bar represents the mean and standard errors for each RIL for which there were replicated observations. For DCV, survival days postinfection was measured. For the sigma virus, the proportion of flies that were paralysed after CO_2_ exposure was measured.

We observed a high level of genetic variation in resistance to both viruses (Fig. 1). For DCV, 77% of the estimated variance in the survival times of infected flies between replicate vials is genetic (i.e. explained by RIL, as calculated using eqn (2); 95% CI: 69–83%). For the sigma virus, 74% of the variance in infection rates was genetic (95% CI: 63–81%).

### Resistance to DCV is controlled by two major‐effect loci that together explain 89% of the genetic variance

We characterized the genetic architecture of DCV resistance by identifying QTL. We regressed our resistance phenotype on the RIL genotypes at 10 kb intervals across the genome and recorded the LOD score. We then repeated this on permuted data to determine the genomewide significance threshold.

**Figure 2:**
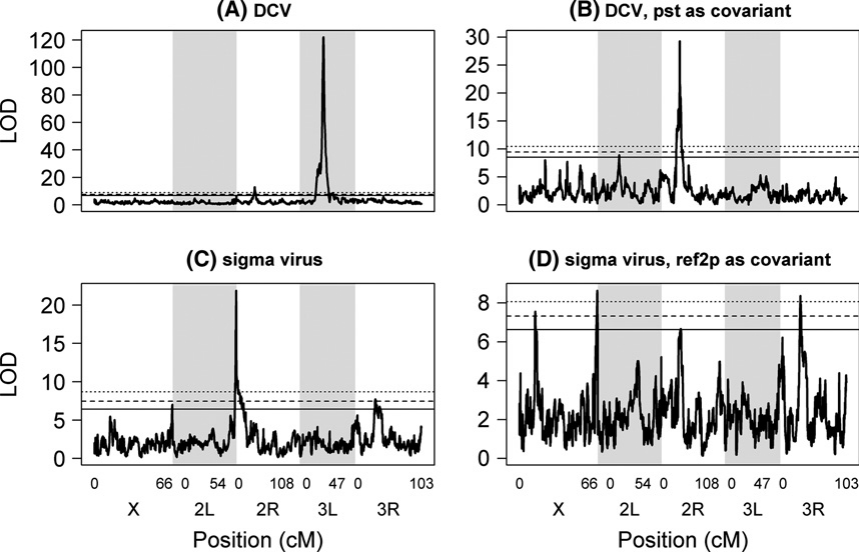
Quantitative trait loci affecting resistance to viral infection in *D. melanogaster*. QTL were mapped that affect resistance to DCV (Panel A) and the sigma virus (Panel C). The analyses were repeated with the genotype at the resistance genes *pastrel* and *ref(2)P* included as covariates (Panels B and D). The horizontal lines are genomewide significance thresholds obtained by permuting the phenotypic data over the RILs (solid line: *P *=* *0.05, dashed line: *P *=* *0.01, dotted line: *P *=* *0.001).

We observed a single major QTL on the left arm of the third chromosome (Fig. 2A). This was extremely significant, with a LOD score of 122 (genomewide significance: *P *<* *0.0005). To localize the QTL more precisely, we performed interval mapping around the mapped QTL and used a Bayesian approach to obtain a 95% credible interval on the QTL location. The resulting 40‐kB region contains nine genes (Table 1; Table S1, Supporting information) including *pastrel (pst)*, which is known to contain a major‐effect polymorphism associated with resistance to DCV (Magwire *et al*. 2012). This gene is therefore very likely causing our QTL.

**Table 1:** Position and credible intervals for the QTL associated with virus resistance. QTL are labelled with putative causative genes or chromosome arm and genetic position

	Chromosome	Peak position (kB)^b^	Bayesian 95% CI (kB)^b^	LOD drop CI (kB)^b^^,^ c	Size (kB)^d^	Genes^d^	*P* a
DCV							
QTL1 – *pst*	3L	7360	7350–7390	7330–7410	40	9	<0.0005
QTL2 – 2R69	2R	9890	9880–9910	9860–9930	30	2	<0.0005
QTL3 – 2L18	2L	5890	5790–6150	5710–6250	360	65	<0.03
Sigma virus							
QTL1 – *ref(2)P*	2L	19 680	19 520–19 900	19 500–20 040	380	34	<0.0005
QTL2 – X65	X	21 730	21420–22310	21 260–22 380	890	30	<0.0005
QTL3 – 3R64	3R	14 590	14 450–14 860	14 410–14 950	410	52	0.001
QTL4 – X13	X	5680	5600–5740	5590–5970	140	23	0.0035
QTL5 – 2R70	2R	10 220	9570–10 350	9470–10 490	780	103	0.0455

a Genomewide significance from permutation.

b Coordinates refer to reference genome version 5.

c Confidence interval based on a LOD drop of 2.

d Size and number of genes within the Bayesian 95% credible interval.

After controlling for the effects of the *pastrel* gene by including it as a covariate in the analysis, we found another highly significant QTL on chromosome arm 2R with a LOD score of 29.0 (Fig. 2B; genomewide significance: *P *<* *0.0005). This QTL included a region of 30 kb, containing just two genes (95% credible intervals on location; Table 1; Table S1, Supporting information). A third minor peak on chromosome 2L (Fig. 2B; LOD = 8.0; genomewide significance: *P *<* *0.03) covered 360 kb and 65 genes (Table 1; Table S1, Supporting information).

**Figure 3:**
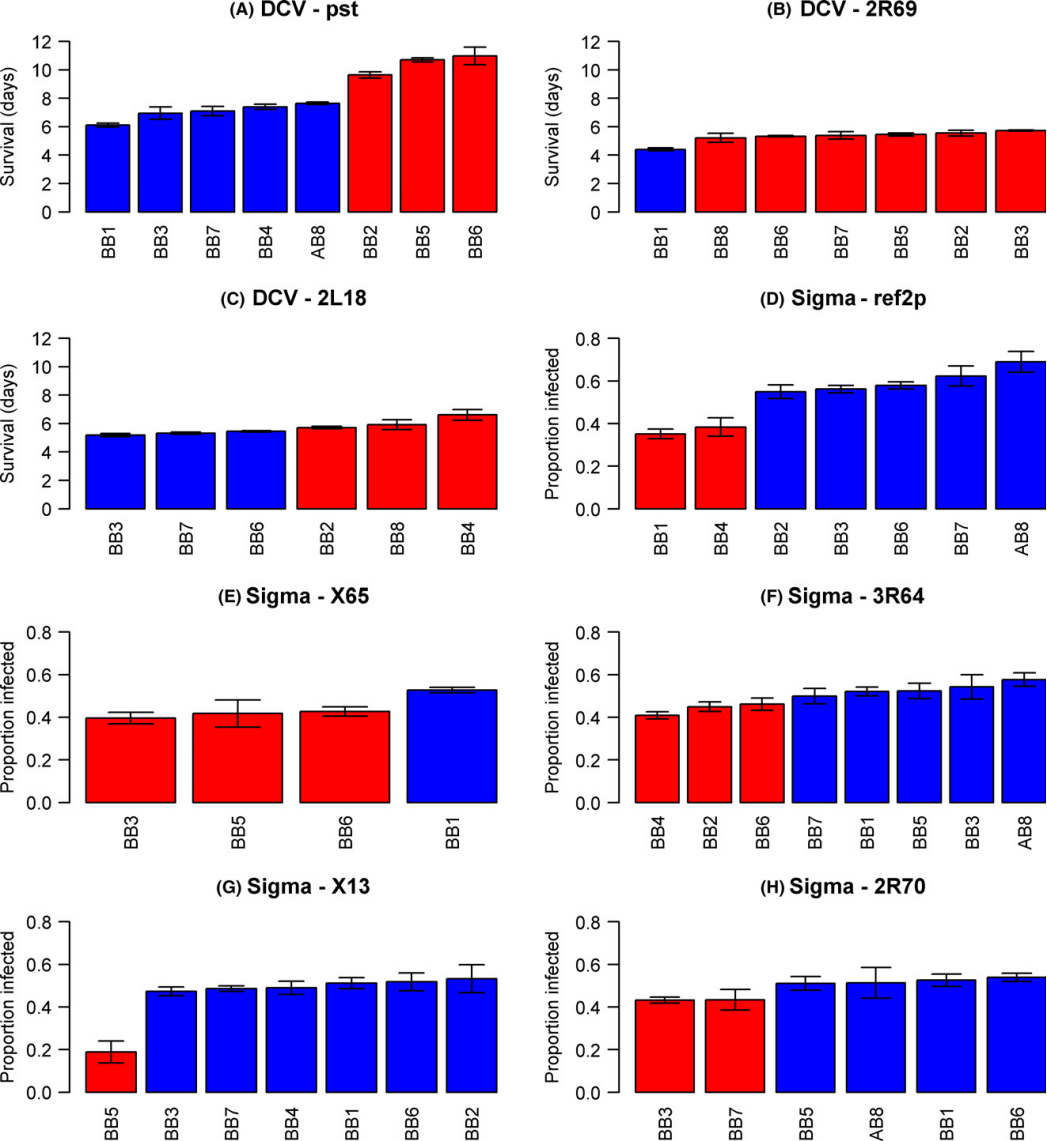
The estimated susceptibility of fly lines carrying the different alleles at each of the QTL affecting DCV and the sigma virus. The alleles were split by maximum likelihood into a resistant class (red) and a susceptible class (blue). Error bars are standard errors of the mean. Panels with less than eight bars are occasions when one or more founders are not represented at a QTL.

Genetic variation in susceptibility could potentially be caused by alleles that are either rare or at intermediate frequencies in populations. The alleles from each of the founders were assigned by maximum likelihood to resistant and susceptible allelic classes. For the *pastrel* QTL, there was a clear division with flies carrying five of the founder alleles dying faster than flies carrying the other three alleles (Fig. 3A, red vs. blue bars). For the QTL on 2R chromosome, one founder was assigned to the susceptible class while the other six founders were assigned to the resistant class (Fig. 3B). For the QTL on 2L chromosome, three founders each were assigned to the resistant and susceptible classes (Fig. 3C). Therefore, the polymorphisms underlying each QTL are at appreciable frequencies among the eight fly lines that founded the mapping population and are unlikely to be rare in nature.

The two main QTL we identified have a large phenotypic effect (Table 2). Flies carrying the resistant allele of *pastrel* die over five days later than flies carrying the resistant allele, while the QTL on chromosome 2R increases survival times by 2.5 days. The QTL also explain most of the genetic variance in the mapping population – 77.8% of the genetic variance in DCV resistance among the RILs is explained by the *pastrel* QTL, and an additional 11.3% of the genetic variance is explained by the QTL on chromosome 2R. Only 0.7% of the genetic variance is explained by the QTL on chromosome 2L (Table 2). Therefore, resistance to DCV is controlled in a near‐Mendelian fashion by two major‐effect loci.

**Table 2:** Effect size and proportion of variance in virus resistance explained by each QTL

Locus^b^	Proportion variance explained (%)	Effect size^c^	Effect size 95% CI	*P* a
DCV				
QTL1 – *pst*	77.8	5.2 days	4.9–5.5	<0.0001
QTL2 – 2R69	11.3	2.5 days	2.0–3.0	<0.0001
QTL3 – 2L18	0.7	0.7 days	0.4–1.1	<0.0001
Sigma				
QTL1 – *ref(2)P*	23.6	30.9%	25.2–36.2	<0.0001
QTL2 – X65	5.0	13.3%	8.4–18.2	<0.0001
QTL3 – 3R64	5.9	11.6%	6.8–16.5	<0.0001
QTL4 – X13	4.5	38.5%	24.4–51.2	<0.0001
QTL5 – 2R70	3.5	10.8%	6.2–15.8	<0.0001

a Posterior probability from MCMCglmm.

b The QTL are named by the chromosome followed by the genetic map position unless the likely causative gene is known.

c Effect size days DCV, % infected sigma.

### Resistance to the sigma virus is controlled by multiple genes of varying effect

We repeated the QTL mapping for sigma virus resistance and found a highly significant peak on the left arm of the second chromosome (Fig. 2C; genomewide significance: *P *<* *0.0005). Again, we performed local interval mapping and calculated a 95% credible interval (Table 1), and this defined a region of 380 kB which contained *c*. 34 genes (Table S1, Supporting information). The recombinants in this region were assigned to founder genotypes, and there was a clear division with two resistant alleles and five susceptible alleles (Fig. 3D).

The gene *ref(2)P*, which contains a known polymorphism associated with resistance to sigma virus (Contamine *et al*. 1989; Wayne *et al*. 1996; Bangham *et al*. 2007), is located within this QTL. As the specific mutation in *ref(2)P* that causes resistance is known (Wayne *et al*. 1996; Bangham *et al*. 2007), we were able to genotype the founder lines by PCR. We found that the resistant QTL alleles had the resistant allele of *ref(2)P*, and the susceptible QTL alleles had the susceptible allele of *ref(2)P*. Therefore, this QTL is very likely caused by *ref(2)P*.

To identify additional loci affecting sigma virus resistance, we repeated the genome scan but used *ref(2)P* allele class as a covariate. This analysis found four additional significant peaks (Fig. 2D). These are found across three different chromosome arms, and the size of the 95% confidence intervals ranges from 140 to 890 kB with one of the QTL containing just 23 genes (Table 1, Table S1, Supporting information).

For each QTL, we assigned the alleles coming from the different lines that founded the mapping population to a resistant or susceptible allelic class (Fig. 3). Of the five sigma QTL (including *ref(2)P*), three had a minor allele that was present in more than one of the eight founder lines (Fig. 3). Therefore, the alleles we are identifying are mostly at an intermediate frequency in nature.

All of the QTL had an appreciable effect on infection rates (Fig. 3, Table 2). The largest effect is from the X13 QTL, which reduced infection rates by 38%. The two resistant alleles of *ref(2)P* are associated with a 30.9% drop in the infection rate. At the other extreme, our smallest effect QTL is the 2R70 QTL, but even this causes a 11% reduction in infection rates.

In combination, our QTL explained 42.5% of the genetic variance among the RILs. Individually, each of our QTL explained from 3.5% to 23.6% of the genetic variance among the RILs, with the *ref(2)P* QTL explaining more of the variance than any of the other loci (Table 2). Therefore, there is still a substantial amount of unexplained genetic variation. In our mapping population, even rare alleles will be pushed to intermediate frequencies, so it is probably that this is caused by loci of small effect.

### QTL effects are independent

In the DSPR population, there can be nonrandom associations between unlinked loci (Corbett‐Detig *et al*. 2013), and so a QTL at one position in the genome could give rise to spurious associations elsewhere. To guard against this, we performed two further analyses. First, we tested for linkage disequilibrium among the significant QTL with Fisher's exact tests. For the QTL associated with sigma virus resistance, most of the loci were not in linkage disequilibrium, but the X65 QTL was associated with the *ref(2)P*, X13, and 2R70 QTL, and the 3R64 QTL was associated with the 2R70 QTL (Table S1, Supporting information). For the three QTL associated with resistance to DCV, *pst* was not in linkage disequilibrium with the 2R69 QTL (*P *=* *0.449) nor with the 2L18 QTL (*P *=* *0.791), and 2R69 is not linked to 2L18 (*P *=* *0.280). Second, we tested for independence of the effect of each QTL on resistance with a general linear mixed model (the model used to estimate effect sizes, see Methods); the type II *P* values from this model give the significance of each QTL taking into accounting all the other loci. For both sigma virus and DCV, all the QTL had a significant effect (Table 2).

### A modifier locus alters the effect of a QTL affecting resistance

**Figure 4:**
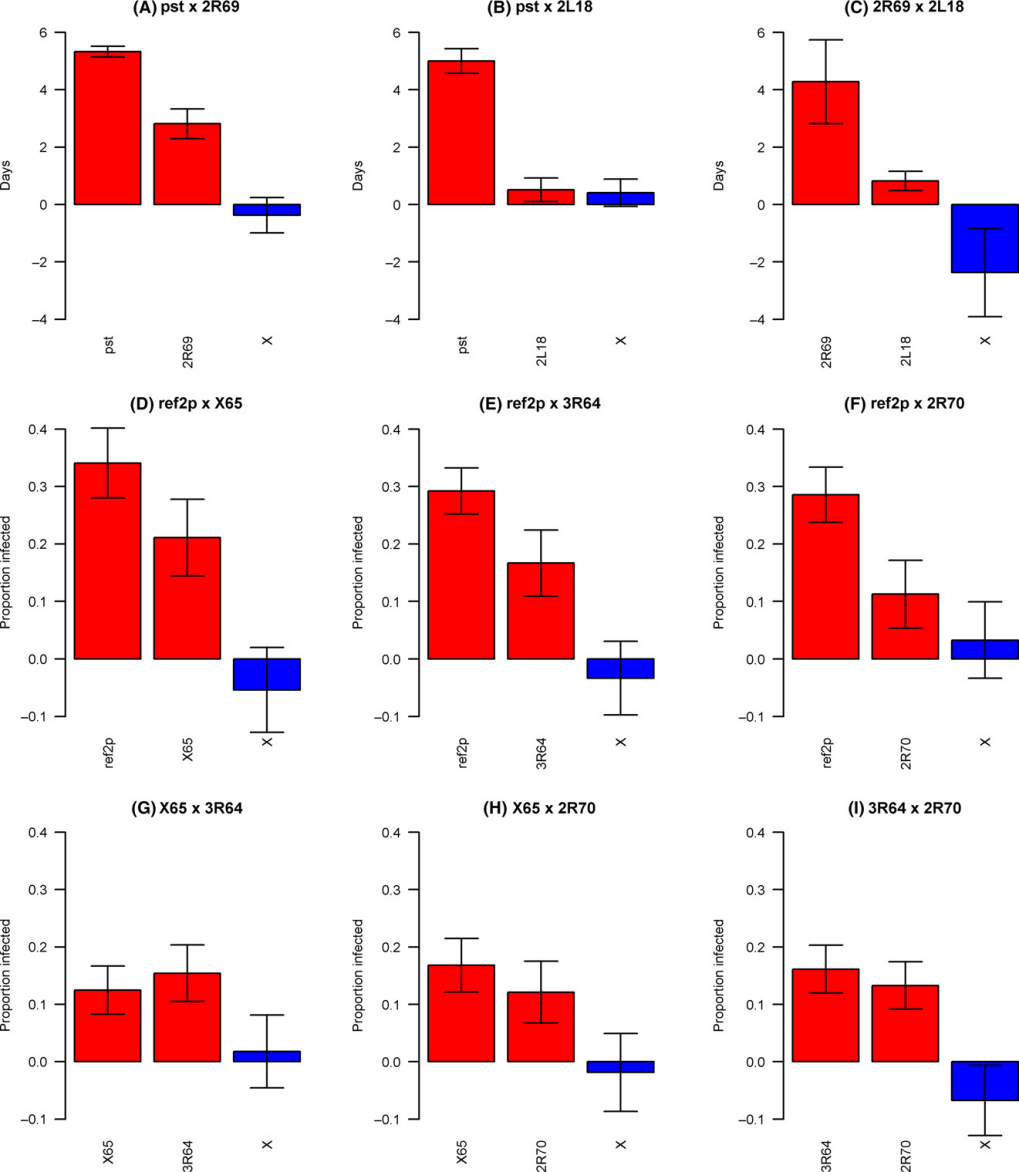
No evidence of pairwise epistasis between QTL affecting virus resistance. For each pair of QTL affecting each virus, the two left bars (red) are the main effects of each locus, and right bar (blue) is the effect of the interaction between the alleles. The bars are standard errors. X labels refer to the interaction term. (A–C) – DCV, (D–I) – sigma virus. Pairs of QTL where the interaction could not be estimated due to missing genotype combinations are not shown.

We took two approaches to test whether genes affecting virus resistance interact epistatically, such that the effect of a locus on the virus depended on the genotype elsewhere in the genome. First, we tested whether the QTL detected above on the basis of their main effects interact. There was no evidence of pairwise epistasis between the three QTL affecting DCV, or six pairwise combinations of QTL affecting the sigma virus (Fig. 4, Table S2, Supporting information). We were not able to test for epistatic effects of the X13 QTL, because we only have 18 resistant RIL (BB5 parent) and many genotype combinations were rare or missing.

**Figure 5:**
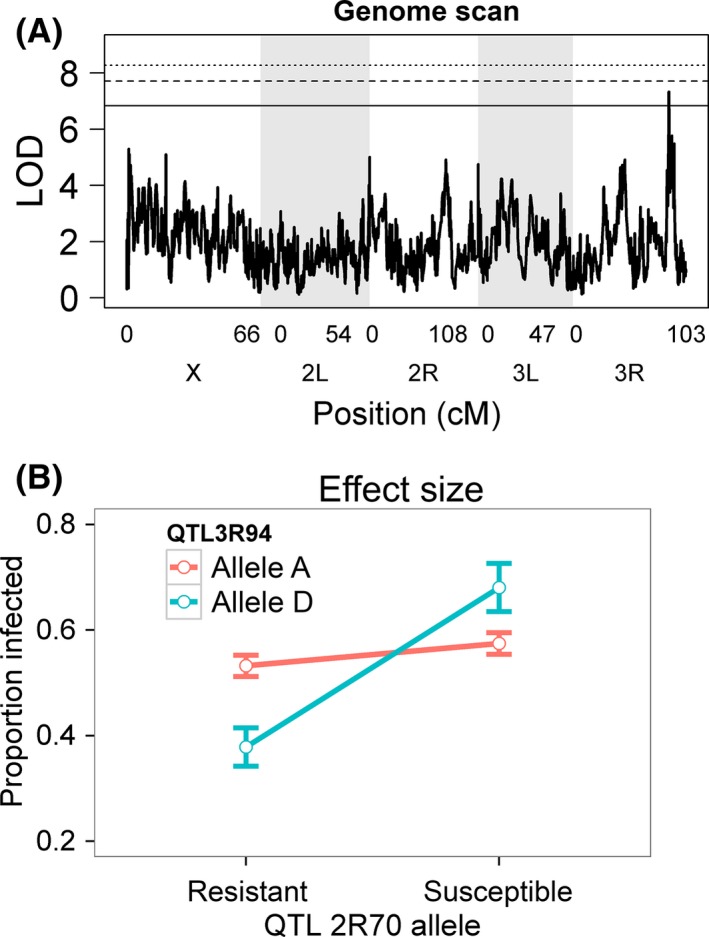
A modifier locus alters the effect of a QTL affecting resistance. Quantitative trait loci that epistatically modify the effect of the 2R70 QTL on resistance to sigma virus infection. (A) QTL were identified by a genome scan looking for interaction of each genome region with the 2R70 QTL. The horizontal lines are genomewide significance thresholds obtained by permuting the phenotypic data over the RILs (solid line: *P *=* *0.05, dashed line: *P *=* *0.01, dotted line: *P *=* *0.001). (B) The epistatic effect on sigma virus resistance of QTL identified by epistasis genome scans. For each QTL, the alleles were split by maximum likelihood into a resistant class and a susceptible class. Values are estimated susceptibility of fly lines carrying the different alleles. Error bars are standard errors of the mean. Results of similar analyses with the other QTL did not identify significant interactions and are not shown.

Genes that have epistatic effects can be difficult to detect on the basis of their main effects, especially if their phenotypic effect is reversed in different genetic backgrounds. Therefore, we mapped additional QTL that modify the effects of the QTL detected above. To do this, we ran genome scans including the genotype of the known QTL as a covariate and examined its interaction with the RIL genotype at 10kB intervals across the genome. We identified a QTL at position 23 670 kb on the right arm of the third chromosome (*P* = 0.024; LOD drop CI: 23 600–23 740 kb; Fig. 5A). This locus alters the effect of the 2R70 QTL allele, such that the allele that made flies more resistant instead makes them more susceptible (Fig. 5B). The scans for the other four sigma QTL and the three DCV QTL did not identify any loci that modified their effects.

## Discussion

We found extensive genetic variation in resistance to two viruses that naturally infect *D. melanogaster* in the wild. We then mapped the genes causing this variation using the *Drosophila* multiparent advanced intercross population, which gives us far greater statistical power to detect genotype–phenotype associations than our previous association studies involving these viruses. For DCV the genetic architecture was near‐Mendelian, we identified a major‐effect locus that increased survival times by about 81% and explained 77.8% of the genetic variation in resistance and another large‐effect QTL that led to 39% increase in survival times and explained 11.3% of the genetic variation. For the sigma virus, on the other hand, we identified five QTL that all had a substantial effect size, causing 10–50% drops in the infection rates. We found no evidence of epistatic interactions among these QTL, meaning that the effect of a locus on susceptibility did not depend on the genotype at other QTL. However, we did identify a modifier locus that reversed the effect of a QTL on sigma virus resistance. Additionally, the QTL we identified are specific to the two different viruses, with no evidence of cross‐resistance.

One explanation of the ‘missing heritability’ in association studies is that much of the variation is caused by rare major‐effect alleles, but we found little evidence to support this. An advantage of our approach is that we can detect rare variants if they are found in the eight lines used to found our mapping population. We find that the DSPR panel has similar levels of genetic variation as in natural populations (Magwire *et al*. 2012). Therefore, if rare variants are the cause of the high genetic variance of this trait we must have some of these alleles in our sample. This is not unexpected, as if rare variants contribute much genetic variance to natural populations then there must be many of them. However, it appears likely that most of the genetic variance in virus resistance in *Drosophila* tends to be caused by alleles at an appreciable frequency in the population. Of the eight identified QTL, seven had more than four founders, and in five of these, both QTL alleles were present in multiple founder lines. Nonetheless, our previous work has identified a rare major‐effect genetic variant in a gene called *Ge‐1* that confers resistance to the sigma virus (Cao *et al*. 2016). Therefore, while such rare variants exist, our data provide little support for the hypothesis that they are the main cause of genetic variation.

We have previously performed genomewide association studies to investigate resistance to the DCV and the sigma virus. These experiments, which used a panel of inbred lines with complete genome sequences (the DGRP panel), are expected to have less statistical power than the analyses presented here. This is largely due to the smaller number of statistical tests performed during QTL mapping meaning that the correction for multiple testing is less severe. As a consequence of this, we have been able to identify numerous additional loci affecting virus resistance. This is most striking for the sigma virus, where our previous work identified just a single locus compared to the five associations reported here. Therefore, we have a far more comprehensive picture of the genetic architecture of virus resistance in *Drosophila melanogaster*.

Resistance to DCV is controlled by a very small number of genes, with two loci accounting for the large majority of the genetic variance. Sigma virus resistance is controlled by five QTL and there is a larger proportion of unexplained genetic variation, but the loci we identified nonetheless had a substantial effect on susceptibility. Overall, our results are consistent with the pattern that variation in viral resistance in *Drosophila* is often affected by major‐effect genes (Bangham *et al*. 2007; Magwire *et al*. 2011, 2012; Martins *et al*. 2014). The other group of natural parasites that is well‐studied in *D. melanogaster* is parasitoid wasps, and here, a few major‐effect loci control resistance (Poirie *et al*. 2000; Hita *et al*. 2006). Resistance to bacteria is possibly more polygenic (Lazzaro *et al*. 2004, 2006), although this may be because true co‐evolved bacterial pathogens of flies have not been isolated. Overall, data from *Drosophila* support the suggestion that the genetic architecture of susceptibility to infectious diseases may often be simpler than the genetic architecture of susceptibility to noncommunicable diseases (Pritchard 2001; Hill 2012).

Mapping genes controlling virus resistance can provide new insights into host–virus interactions and antiviral immunity. Two of the QTL we found contain genes that are known to control resistance to these viruses – *Pastrel,* which is associated with DCV resistance (Magwire *et al*. 2012), and *ref(2)p*, which is associated with sigma virus resistance (Contamine *et al*. 1989; Wayne *et al*. 1996; Bangham *et al*. 2007). A third major‐effect polymorphism in the gene *CHKov1* (Magwire *et al*. 2011) was fixed for the resistant allele in this population. Another gene associated with sigma virus resistance, Ge‐1 (Cao *et al*. 2016), was fixed for the susceptible allele in the DSPR population. The novel QTL we identified are as small as 30kB, and contain as few as two genes, so future research can use the genetic tools available in *Drosophila* to identify the other genes causing viral resistance. Resistance to viruses can evolve through changes in either the immune system (Felix *et al*. 2011) or host factors that are used by the virus during its replication cycle such as the receptor used to enter cells (Karlsson *et al*. 2003). The antiviral immune response of insects is poorly understood compared to antibacterial and antifungal immunity (Kemp & Imler 2009); therefore, this can lead to new insights into the evolution of resistance to infection, as well as the mechanisms of virus interaction with hosts.

Models of co‐evolution commonly assume epistasis between alleles (Bergelson *et al*. 2001; Fenton & Brockhurst 2007), but we found that all the QTL we first identified had independent effects on resistance. However, loci that epistatically modify resistance may be hard to identify from their main effects. When we scanned for additional QTL that modify the effect of the first set of QTL we identified, we were able to identify an additional locus that reversed the effect of a resistance gene. Wilfert and Schmid‐Hempel (Wilfert & Schmid‐Hempel 2008) reviewed published studies that have identified QTL for host resistance in animals and plants, and found that epistatic interactions were presented in the majority of cases and were responsible for a substantial amount of the explained variance. Our results suggest that in our system epistatic interactions do occur, but they are unlikely to have such pervasive effects.

The heritability that remains unexplained in our study is probably caused by minor‐effect genes. The identified QTL are responsible for a large proportion of the genetic variation in virus resistance. For DCV, the three QTL explained 90% of the variation, and for the sigma virus, the five QTL explained 42.5%. As discussed above, rare alleles of moderate and large effect should be detectable, because the panel is founded by eight parents, pushing rare alleles to intermediate frequencies (King *et al*. 2012). In addition, widespread allelic heterogeneity should not affect the detection of QTL (King *et al*. 2014). Ruling out these two possible causes for the missing heritability, the most likely explanation is the presence of many minor‐effect loci (Yang *et al*. 2010; Rockman 2012). Alternatively, part of the missing heritability may be caused by unknown loci that interact epistatically (Huang *et al*. 2012; Zuk *et al*. 2012), although the lack of epistasis among the loci we did detect suggests this may be less likely.

Our work focused on just a single isolate of each virus. This is important, as resistance genes may have specific effects on specific virus genotypes, and this may be important in the maintenance of genetic variation and co‐evolution. For example, during the late 20th century genotypes of the sigma virus that were not affected by the resistant allele of *ref(2)P* spread through European populations of *D. melanogaster* (Wilfert & Jiggins 2013). Further work could extend this analysis to understand how genetic variation in the virus population interacts with genetic variation in the host population. In the future, an important task will to be to identify the genes underlying resistance. We have inspected the genes within the QTL are there are no obvious candidates, so this will probably involve additional genetic mapping to identify the causative loci.

In conclusion, the use of multiparent advanced intercross populations here was a powerful tool to investigate the genetic architecture of virus resistance, making great advances from our previous study using the DGRP (Magwire *et al*. 2012). First, because we have higher statistical power we were able to identify six additional QTL, most of which had substantial phenotypic effects. Therefore, the major‐effect genes commonly assumed by theory do appear to be common in nature. Second, we were able to show a lack of epistatic interactions among the major identified QTL, and identify an additional QTL that reverses the effect of one of the initially identified QTL. Overall, this suggests that strong epistatic effects are probably not a major cause of genetic variation virus resistance in *Drosophila*. Finally, several of the major‐effect QTL were found in more than one of the eight founders of our mapping population, indicating that genetic variation is not being caused by large numbers of rare variants of large effect.

R.C., C.C., and F.J. designed research, analysed data and wrote the manuscript. R.C., C.C., C.B., and J.D. performed research.

## Data accessibility

Phenotype raw data and all scripts used in the analyses are available at Cambridge data repository (https://www.repository.cam.ac.uk/handle/1810/255877). The genetic variants data for the RILs and all analyses tools used are accessible at http://wfitch.bio.uci.edu/~dspr/.

## Supporting information


**Table S1** List of genes present in each identified QTL.

**Table S2** Test of linkage disequilibrium among loci on the RIL.
**Table S3** Analysis of deviance table for models testing for epistasis.

## References

[mec13769-bib-0001] Arnold PA , Johnson KN , White CR (2013) Physiological and metabolic consequences of viral infection in *Drosophila melanogaster* . The Journal of Experimental Biology, 216, 3350–3357. 23685974 10.1242/jeb.088138

[mec13769-bib-0002] Bangham J , Obbard DJ , Kim KW , Haddrill PR , Jiggins FM (2007) The age and evolution of an antiviral resistance mutation in *Drosophila melanogaster* . Proceedings Biological Sciences/The Royal Society, 274, 2027–2034. 10.1098/rspb.2007.0611 PMC1914336 17550883

[mec13769-bib-0003] Bangham J , Knott SA , Kim K‐W , Young RS , Jiggins FM (2008) Genetic variation affecting host‐parasite interactions: major‐effect quantitative trait loci affect the transmission of sigma virus in *Drosophila melanogaster* . Molecular Ecology, 17, 3800–3807. 18665899 10.1111/j.1365-294X.2008.03873.x

[mec13769-bib-0004] Bansal V , Libiger O , Torkamani A , Schork NJ (2010) Statistical analysis strategies for association studies involving rare variants. Nature Reviews. Genetics, 11, 773–785. 10.1038/nrg2867 PMC3743540 20940738

[mec13769-bib-0005] Bennett KE , Flick D , Fleming KH *et al* (2005) Quantitative trait loci that control dengue‐2 virus dissemination in the mosquito *Aedes aegypti* . Genetics, 170, 185–194. 15781707 10.1534/genetics.104.035634 PMC1449711

[mec13769-bib-0006] Bergelson J , Dwyer G , Emerson JJ (2001) Models and data on plant‐enemy coevolution. Annual Review of Genetics, 35, 469–499. 10.1146/annurev.genet.35.102401.090954 11700291

[mec13769-bib-0007] Broman KW , Sen S (2009) A Guide to QTL Mapping with R/qtl. Springer, New York.

[mec13769-bib-0008] Brun G , Plus N (1980) The viruses of Drosophila In: The Genetics and Biology of Drosophila(eds Ashburner M, Wright TRF), pp. 625–702. Academic Press, New York.

[mec13769-bib-0009] Buckler ES , Holland JB , Bradbury PJ *et al* (2009) The genetic architecture of maize flowering time. Science, 325, 714–718. 19661422 10.1126/science.1174276

[mec13769-bib-0010] Cao C , Magwire MM , Bayer F , Jiggins FM (2016) A polymorphism in the processing body component Ge‐1 controls resistance to a naturally occurring Rhabdo virus in *Drosophila* . PLoS Pathogens, 12, e1005387. 26799957 10.1371/journal.ppat.1005387 PMC4723093

[mec13769-bib-0011] Christian P (1987) Studies of Drosophila C and A viruses in Australian populations of Drosophila melanogaster. Australian National University.

[mec13769-bib-0012] Churchill GA , Doerge RW (1994) Empirical threshold values for quantitative trait mapping. Genetics, 138, 963–971. 7851788 10.1093/genetics/138.3.963 PMC1206241

[mec13769-bib-0013] Churchill GA , Airey DC , Allayee H *et al* (2004) The collaborative cross, a community resource for the genetic analysis of complex traits. Nature Genetics, 36, 1133–1137. 15514660 10.1038/ng1104-1133

[mec13769-bib-0014] Clark B (1976) The ecological genetics of host‐parasite relationships. Symposia of the British Society for Parasitology, 14, 87–103.

[mec13769-bib-0015] Contamine D , Petitjean AM , Ashburner M (1989) Genetic resistance to viral infection: the molecular cloning of a *Drosophila* gene that restricts infection by the Rhabdo virus sigma. Genetics, 123, 525–533. 2557263 10.1093/genetics/123.3.525 PMC1203824

[mec13769-bib-0016] Cooke GS , Hill AV (2001) Genetics of susceptibility to human infectious disease. Nature Reviews Genetics, 2, 967–977. 10.1038/35103577 11733749

[mec13769-bib-0017] Corbett‐Detig RB , Zhou J , Clark AG , Hartl DL , Ayroles JF (2013) Genetic incompatibilities are widespread within species. Nature, 504, 135–137. 24196712 10.1038/nature12678 PMC4844467

[mec13769-bib-0018] Coulon P , Contamine D (1982) Role of the *Drosophila* genome in sigma virus multiplication. II. Host spectrum variants among the haP mutants. Virology, 123, 381–392. 7179741 10.1016/0042-6822(82)90271-9

[mec13769-bib-0019] Do R , Kathiresan S , Abecasis GR (2012) Exome sequencing and complex disease: practical aspects of rare variant association studies. Human Molecular Genetics, 21, R1–R9. 22983955 10.1093/hmg/dds387 PMC3459641

[mec13769-bib-0020] Felix M‐A , Ashe A , Piffaretti J *et al* (2011) Natural and experimental infection of *Caenorhabditis* nematodes by novel viruses related to nodaviruses. PLoS Biology, 9, 1–14. 10.1371/journal.pbio.1000586 PMC3026760 21283608

[mec13769-bib-0021] Fenton A , Brockhurst MA (2007) Epistatic interactions alter dynamics of multilocus gene‐for‐gene coevolution. PLoS ONE, 2, e1156. 17989777 10.1371/journal.pone.0001156 PMC2065793

[mec13769-bib-0022] Hadfield JD (2010) MCMC methods for multi‐response generalized linear mixed models: the MCMCglmm R package. Journal of Statistical Software, 33, 1–22. 20808728 PMC2929880

[mec13769-bib-0023] Hill AV (2012) Evolution, revolution and heresy in the genetics of infectious disease susceptibility. Philosophical Transactions of the Royal Society of London. Series B, Biological Sciences, 367, 840–849. 22312051 10.1098/rstb.2011.0275 PMC3267114

[mec13769-bib-0024] Hita M , Espagne E , Lemeunier F *et al* (2006) Mapping candidate genes for *Drosophila melanogaster* resistance to the parasitoid wasp *Leptopilina boulardi* . Genetical Research, 88, 81–91. 17125583 10.1017/S001667230600841X

[mec13769-bib-0025] Huang W , Richards S , Carbone MA *et al* (2012) Epistasis dominates the genetic architecture of *Drosophila* quantitative traits. Proceedings of the National Academy of Sciences of the United States of America, 109, 15553–15559. 22949659 10.1073/pnas.1213423109 PMC3465439

[mec13769-bib-0026] Jaenike J (1978) An hypothesis to account for the maintenance of sex within populations. Evolutionary Theory, 3, 191–194.

[mec13769-bib-0027] Johnson KN , Christian PD (1999) Molecular characterization of *Drosophila C virus* isolates. Journal of Invertebrate Pathology, 73, 248–254. 10222177 10.1006/jipa.1998.4830

[mec13769-bib-0028] Jousset FX , Plus N , Croizier G , Thomas M (1972) Existence in *Drosophila* of 2 groups of picornavirus with different biological and serological properties. Comptes Rendus Hebdomadaires des Seances de l'Academie des Sciences Serie D: Sciences Naturelles, 275, 3043–3046. 4631976

[mec13769-bib-0029] Karlsson F , Borrebaeck CAK , Nilsson N , Malmborg‐Hager AC (2003) The mechanism of bacterial infection by filamentous phages involves molecular interactions between TolA and phage protein 3 domains. Journal of Bacteriology, 185, 2628–2634. 12670988 10.1128/JB.185.8.2628-2634.2003 PMC152608

[mec13769-bib-0030] Kemp C , Imler JL (2009) Antiviral immunity in *Drosophila* . Current Opinion in Immunology, 21, 3–9. 19223163 10.1016/j.coi.2009.01.007 PMC2709802

[mec13769-bib-0031] King EG , Merkes CM , McNeil CL *et al* (2012) Genetic dissection of a model complex trait using the *Drosophila* synthetic population resource. Genome Research, 22, 1558–1566. 22496517 10.1101/gr.134031.111 PMC3409269

[mec13769-bib-0032] King EG , Sanderson BJ , McNeil CL , Long AD , Macdonald SJ (2014) Genetic dissection of the *Drosophila melanogaster* female head transcriptome reveals widespread allelic heterogeneity. PLoS Genetics, 10, e1004322. 24810915 10.1371/journal.pgen.1004322 PMC4014434

[mec13769-bib-0033] Kover PX , Valdar W , Trakalo J *et al* (2009) A Multiparent advanced generation inter‐cross to fine‐map quantitative traits in *Arabidopsis thaliana* . PLoS Genetics, 5, e1000551. 19593375 10.1371/journal.pgen.1000551 PMC2700969

[mec13769-bib-0035] Lander ES , Botstein D (1989) Mapping mendelian factors underlying quantitative traits using RFLP linkage maps. Genetics, 121, 185–199. 2563713 10.1093/genetics/121.1.185 PMC1203601

[mec13769-bib-0036] Lazzaro BP , Sceurman BK , Clark AG (2004) Genetic basis of natural variation in *D. melanogaster* antibacterial immunity. Science, 303, 1873–1876. 15031506 10.1126/science.1092447

[mec13769-bib-0037] Lazzaro BP , Sackton TB , Clark AG (2006) Genetic variation in *Drosophila melanogaster* resistance to infection: a comparison across bacteria. Genetics, 174, 1539–1554. 16888344 10.1534/genetics.105.054593 PMC1667071

[mec13769-bib-0038] Long AD , Macdonald SJ , King EG (2014) Dissecting complex traits using the *Drosophila* synthetic population resource. Trends in Genetics, 30, 488–495. 25175100 10.1016/j.tig.2014.07.009 PMC4253597

[mec13769-bib-0039] Longdon B , Hadfield JD , Webster CL , Obbard DJ , Jiggins FM (2011) Host phylogeny determines viral persistence and replication in novel hosts. PLoS Pathogens, 7, e1002260. 21966271 10.1371/journal.ppat.1002260 PMC3178573

[mec13769-bib-0040] Longdon B , Wilfert L , Jiggins FM (2012) The sigma viruses of *Drosophila* In: Rhabdoviruses: Molecular Taxonomy, Evolution, Genomics, Ecology, Cytopathology and Control (eds Dietzgen R, Kuzmin I), pp. 117–132. Caister Academic Press, Kuzmin.

[mec13769-bib-0041] Longdon B , Hadfield JD , Day JP *et al* (2015) The causes and consequences of changes in virulence following pathogen host shifts. PLoS Pathogens, 11, e1004728. 25774803 10.1371/journal.ppat.1004728 PMC4361674

[mec13769-bib-0042] Mackay TF (2014) Epistasis and quantitative traits: using model organisms to study gene‐gene interactions. Nature Reviews Genetics, 15, 22–33. 10.1038/nrg3627 PMC3918431 24296533

[mec13769-bib-0043] Mackay TF , Stone EA , Ayroles JF (2009) The genetics of quantitative traits: challenges and prospects. Nature Reviews Genetics, 10, 565–577. 10.1038/nrg2612 19584810

[mec13769-bib-0044] Magwire MM , Bayer F , Webster CL , Cao C , Jiggins FM (2011) Successive increases in the resistance of *Drosophila* to viral infection through a transposon insertion followed by a duplication. PLoS Genetics, 7, e1002337. 22028673 10.1371/journal.pgen.1002337 PMC3197678

[mec13769-bib-0045] Magwire MM , Fabian DK , Schweyen H *et al* (2012) Genome‐wide association studies reveal a simple genetic basis of resistance to naturally coevolving viruses in *Drosophila melanogaster* . PLoS Genetics, 8, e1003057. 23166512 10.1371/journal.pgen.1003057 PMC3499358

[mec13769-bib-0046] Manolio TA , Collins FS , Cox NJ *et al* (2009) Finding the missing heritability of complex diseases. Nature, 461, 747–753. 19812666 10.1038/nature08494 PMC2831613

[mec13769-bib-0047] Martinez J , Longdon B , Bauer S *et al* (2014) Symbionts commonly provide broad spectrum resistance to viruses in insects: a comparative analysis of wolbachia strains. PLoS Pathogens, 10, e1004369. 25233341 10.1371/journal.ppat.1004369 PMC4169468

[mec13769-bib-0048] Martins NE , Faria VG , Nolte V *et al* (2014) Host adaptation to viruses relies on few genes with different cross‐resistance properties. Proceedings of the National Academy of Sciences of the United States of America, 111, 5938–5943. 24711428 10.1073/pnas.1400378111 PMC4000853

[mec13769-bib-0049] Mirabello L , Macari ER , Jessop L *et al* (2014) Whole‐exome sequencing and functional studies identify RPS29 as a novel gene mutated in multicase Diamond‐Blackfan anemia families. Blood, 124, 24–32. 24829207 10.1182/blood-2013-11-540278 PMC4125351

[mec13769-bib-0050] Poirie M , Frey F , Hita M *et al* (2000) *Drosophila* resistance genes to parasitoids: chromosomal location and linkage analysis. Proceedings Biological Sciences/The Royal Society, 267, 1417–1421. 10.1098/rspb.2000.1158 PMC1690685 10983825

[mec13769-bib-0051] Pritchard JK (2001) Are rare variants responsible for susceptibility to complex diseases? American Journal of Human Genetics, 69, 124–137. 11404818 10.1086/321272 PMC1226027

[mec13769-bib-0052] Rockman MV (2012) The QTN program and the alleles that matter for evolution: all that's gold does not glitter. Evolution, 66, 1–17. 22220860 10.1111/j.1558-5646.2011.01486.x PMC3386609

[mec13769-bib-0053] Sorci G , Møller AP , Boulinier T (1997) Genetics of host‐parasite interactions. Trends in Ecology and Evolution, 12, 196–200. 21238038 10.1016/s0169-5347(97)01056-2

[mec13769-bib-0054] Stahl EA , Dwyer G , Mauricio R , Kreitman M , Bergelson J (1999) Dynamics of disease resistance polymorphism at the Rpm1 locus of Arabidopsis. Nature, 400, 667–671. 10458161 10.1038/23260

[mec13769-bib-0055] Teixeira L , Ferreira A , Ashburner M (2008) The bacterial symbiont Wolbachia induces resistance to RNA viral infections in *Drosophila melanogaster* . PLoS Biology, 6, e2. 10.1371/journal.pbio.1000002 PMC2605931 19222304

[mec13769-bib-0056] Tellier A , Brown JKM (2007) Polymorphism in multilocus host‐parasite coevolutionary interactions. Genetics, 177, 1777–1790. 17947440 10.1534/genetics.107.074393 PMC2147965

[mec13769-bib-0057] Thompson JN , Burdon JJ (1992) Gene‐for‐gene coevolution between plants and parasites. Nature, 360, 121–125.

[mec13769-bib-0058] Thornton KR , Foran AJ , Long AD (2013) Properties and modeling of GWAS when complex disease risk is due to non‐complementing, deleterious mutations in genes of large effect. PLoS Genetics, 9, e1003258. 23437004 10.1371/journal.pgen.1003258 PMC3578756

[mec13769-bib-0059] Visscher PM , Brown MA , McCarthy MI , Yang J (2012) Five years of GWAS discovery. American Journal of Human Genetics, 90, 7–24. 22243964 10.1016/j.ajhg.2011.11.029 PMC3257326

[mec13769-bib-0060] Wayne ML , Contamine D , Kreitman M (1996) Molecular population genetics of ref(2)P, a locus which confers viral resistance in *Drosophila* . Molecular Biology and Evolution, 13, 191–199. 8583891 10.1093/oxfordjournals.molbev.a025555

[mec13769-bib-0061] Wilfert L , Jiggins FM (2013) The dynamics of reciprocal selective sweeps of host resistance and a parasite counter‐adaptation in *Drosophila* . Evolution, 67, 761–773. 23461326 10.1111/j.1558-5646.2012.01832.x

[mec13769-bib-0062] Wilfert L , Schmid‐Hempel P (2008) The genetic architecture of susceptibility to parasites. BMC Evolutionary Biology, 8, 187. 18590517 10.1186/1471-2148-8-187 PMC2446395

[mec13769-bib-0063] Woolhouse ME , Webster JP , Domingo E , Charlesworth B , Levin BR (2002) Biological and biomedical implications of the co‐evolution of pathogens and their hosts. Nature Genetics, 32, 569–577. 12457190 10.1038/ng1202-569

[mec13769-bib-0064] Woolhouse ME , Haydon DT , Antia R (2005) Emerging pathogens: the epidemiology and evolution of species jumps. Trends in Ecology and Evolution, 20, 238–244. 16701375 10.1016/j.tree.2005.02.009 PMC7119200

[mec13769-bib-0065] Yampolsky LY , Webb CT , Shabalina SA , Kondrashov AS (1999) Rapid accumulation of a vertically transmitted parasite triggered by relaxation of natural selection among hosts. Evolutionary Ecology Research, 1, 581–589.

[mec13769-bib-0066] Yang J , Benyamin B , McEvoy BP *et al* (2010) Common SNPs explain a large proportion of the heritability for human height. Nature Genetics, 42, 565–569. 20562875 10.1038/ng.608 PMC3232052

[mec13769-bib-0067] Zuk O , Hechter E , Sunyaev SR , Lander ES (2012) The mystery of missing heritability: genetic interactions create phantom heritability. Proceedings of the National Academic Sciences of the United States of America, 109, 1193–1198. 10.1073/pnas.1119675109 PMC3268279 22223662

