# Supplementary material for: The genetic architecture of resistance to virus infection in Drosophila
Source: Mol Ecol. 2016 Aug 26;25(20):5228–41. doi: 10.1111/mec.13769 (PMC5082504; doi:10.1111/mec.13769)
Supplement: Supplementary file 2 — Table S2 Test of linkage disequilibrium among loci on the RIL. Table S3 Analysis of deviance table for models testing for epistasis. [file MEC-25-5228-s002.docx]

**The genetic architecture of resistance to virus infection in *Drosophila***

**Rodrigo Cogni, Chuan Cao, Jonathan P. Day, Calum Bridson, Francis M. Jiggins**

**Supplemental information**

**Table S1.** List of genes present in each identified QTL (attached as Excel workbook)

**Table S2.** Test of linkage disequilibrium among loci on the RIL associated with sigma virus resistance. Simulated P value for Fisher exact test. Significant differences are in italic.

|  | QTL1 – *ref(2)P* | QTL2 – X65 | QTL3 – 3R64 | QTL4 – X13 | QTL5 – 2R70 |
| --- | --- | --- | --- | --- | --- |
| QTL1 – *ref(2)P* | - |  |  |  |  |
| QTL2 – X65 | *0.022* | - |  |  |  |
| QTL3 – 3R64 | 0.073 | 0.235 | - |  |  |
| QTL4 – X13 | 0.143 | *0.017* | 0.230 | - |  |
| QTL5 – 2R70 | 0.096 | *0.036* | *0.018* | >0.999 | - |

**Table S3.** Analysis of deviance table (Type III Wald χ^2^ tests) for models testing for epistasis.

| Factor | χ2 | d.f. | P |
| --- | --- | --- | --- |
| **DCV** |  |  |  |
| (A) *pst* and 2R69 |  |  |  |
| *pst* | 802.3 | 1 | <2.2e^-16^ |
| 2R69 | 29.3 | 1 | 6.058e^-8^ |
| *pst**2R69 | 0.37 | 1 | 0.5441 |
| (B) *pst* and 2L18 |  |  |  |
| *pst* | 136.2 | 1 | 2.2e-^16^ |
| 2L18 | 1.59 | 1 | 0.2076 |
| *pst**2R69 | 0.75 | 1 | 0.3855 |
| (C) 2R69 and 2L18 |  |  |  |
| 2R69 | 8.6 | 1 | 0.0033 |
| 2L18 | 6.0 | 1 | 0.0140 |
| 2R69*2L18 | 2.4 | 1 | 0.1215 |
| **Sigma virus** |  |  |  |
| (D) *ref(2)p* and X65 |  |  |  |
| *ref(2)p* | 31.43 | 1 | 2.071e^-08^ |
| X65 | 9.98 | 1 | 0.00158 |
| *ref(2)p**X65 | 0.53 | 1 | 0.46580 |
| (E) *ref(2)p* and 3R64 |  |  |  |
| *ref(2)p* | 53.0 | 1 | 3.336e^-13^ |
| 3R64 | 8.40 | 1 | 0.00374 |
| *ref(2)p**3R64 | 0.27 | 1 | 0.60453 |
| (F) *ref(2)p* and 2R70 |  |  |  |
| *ref(2)p* | 35.37 | 1 | 2.721e^-09^ |
| 2R70 | 3.66 | 1 | 0.05558 |
| ref(2)p*2R70 | 0.25 | 1 | 0.61851 |
| (G) X65 and 3R64 |  |  |  |
| X65 | 8.77 | 1 | 0.00306 |
| 3R64 | 9.84 | 1 | 0.00171 |
| X65*3R64 | 0.08 | 1 | 0.77650 |
| (H) X65 and 2R70 |  |  |  |
| X65 | 12.91 | 1 | 0.00033 |
| 2R70 | 5.06 | 1 | 0.02451 |
| X65*2R70 | 0.08 | 1 | 0.78278 |
| (I) 3R64 and 2R70 |  |  |  |
| 3R64 | 14.9 | 1 | 0.00011 |
| 2R70 | 10.3 | 1 | 0.00133 |
| 3R64*2R70 | 1.22 | 1 | 0.26953 |
